# Guillain–Barré Syndrome Following Malaria: A Rare Case Report From Rural Ethiopia and Literature Review

**DOI:** 10.1002/ccr3.71503

**Published:** 2025-11-19

**Authors:** Hayatu Awel Abdela, Numeri Husein Kimo, Tamirat Godebo Woyimo

**Affiliations:** ^1^ Department of Internal Medicine, School of Medicine, College of Medicine and Health Sciences Wolkite University Wolkite Ethiopia; ^2^ Department of Pediatrics and Child Health, School of Medicine, College of Medicine and Health Sciences Wolkite University Wolkite Ethiopia; ^3^ Department of Internal Medicine, School of Medicine, College of Medicine and Health Sciences Jimma University Jimma Ethiopia

**Keywords:** acute inflammatory demyelinating polyneuropathy, case report, Ethiopia, Guillain–Barré syndrome, malaria, *Plasmodium vivax*

## Abstract

Guillain–Barré syndrome (GBS) is an acute immune‐mediated polyradiculoneuropathy often triggered by infections. Malaria, a common parasitic disease in endemic regions, has rarely been associated with GBS. We report a case of a 21‐year‐old male from rural Ethiopia who developed GBS following *Plasmodium vivax* malaria. The patient presented with ascending paralysis, areflexia, dysphagia, dysarthria, and autonomic dysfunction. Cerebrospinal fluid (CSF) analysis revealed albumin‐cytologic dissociation, which further strengthens the diagnosis of GBS. He was successfully treated with intravenous immunoglobulin (IVIg) and supportive care. The patient achieved full recovery after 8 weeks from the onset of weakness. This case highlights the importance of considering GBS in patients with recent malaria infection and neurological deficits, particularly in malaria‐endemic regions. The rarity of such cases in the literature and the unique clinical course make this report noteworthy.

**Summary**

- Guillain–Barré syndrome (GBS) is an autoimmune neuropathy frequently triggered by infections.
- Malaria is not a common infection to trigger GBS. We report a case of GBS following *Plasmodium vivax* infection.
- The 21‐year‐old patient developed ascending paralysis, sensory symptoms without signs and autonomic fluctuations, 5 days after *P. vivax* infection.
- He made an uneventful recovery after treatment with IVIG.

## 1 Introduction

Guillain–Barré syndrome (GBS) is an acute inflammatory demyelinating polyneuropathy often triggered by antecedent infections, such as *Campylobacter jejuni* , cytomegalovirus, or Epstein–Barr virus. Malaria, a leading cause of febrile illness in endemic areas, has rarely been implicated as a trigger for GBS [1]. Here, we present a case of GBS following *Plasmodium vivax* malaria, and a literature review focusing on cases of malaria‐associated GBS emphasizing the clinical details, diagnostic challenges, management and outcomes.

## 2 Case Presentation

A 21‐year‐old male from rural Ethiopia presented with a 4‐day history of ascending symmetrical weakness, starting with numbness in fingers and toes, progressing to all limbs and facial muscles, with dysphagia, drooling, dysarthria, urinary retention, hyperesthesia, and back pain. Muscle paralysis peaked by Day‐9. Five days prior, he was diagnosed with *P. vivax* malaria by thick blood smear, after fever, headache, chills, rigors, arthralgia, and myalgia, treated with artemether–lumefantrine, with febrile symptoms resolved. No other triggers (e.g., vaccination, toxins, infections) were reported.

Examination showed dysarthria, impaired gag reflex, symmetrical weakness (upper/lower limbs: 2/5, neck: 3/5 on Medical Research Council scale), absent deep tendon reflexes (Graded 0/4; scale: 0 = absent reflex, 1 = hyporeflex, 2 = normal reflex, 3 = hyperreflex, 4 = clonus) in the knees, ankles, triceps and biceps bilaterally, and autonomic dysfunction (urinary retention, fluctuating blood pressure [120–170/70–115 mmHg], heart rate [80–130 bpm]). Vital capacity (single breath count) was not assessed due to the patient's dysarthria.

## 3 Investigations and Treatment

Cerebrospinal fluid (CSF) analysis on Day‐6 of weakness revealed albuminocytologic dissociation (protein: 86 mg/dL, glucose: 65 mg/dL, no cells). Routine labs and infectious/autoimmune serologies were normal. Nerve conduction studies were unavailable.

The patient received intravenous immunoglobulin (IVIg, 2 g/kg over 5 days) starting Day 9, gabapentin (300 mg TID via nasogastric tube) for pain, and labetalol (20–40 mg per need) for autonomic instability. A nasogastric tube and urinary catheter were inserted, and he was admitted to the intensive care unit (ICU) without intubation.

## 4 Outcome and Follow‐Up

By Day 21, he showed improvement (upper limbs power: 4/5, lower limbs power: 3/5, reduced drooling, audible speech). By discharge on Day 26, he could stand with support, resume oral intake, void independently, and had minimal pain. At 4‐week follow‐up, he fully recovered, resuming normal activities with unremarkable neurological findings (Table 1).

**TABLE 1:** Chronological order of clinical events in post‐malarial Guillain–Barré syndrome.

Event	Day	Clinical details	Key interventions/findings
Malaria diagnosis	5 days prior to weakness	High‐grade fever, headache, chills, rigors, arthralgia, myalgia	*Plasmodium vivax* confirmed; Artemether–lumefantrine initiated
GBS symptom onset	0	Numbness in fingers/toes; ascending symmetrical weakness	
Emergency department visit	4	Dysarthria, impaired gag reflex, areflexia, autonomic instability	Lumbar puncture done Labetalol initiated (20–40 mg per need) Nerve conduction test was not done due to resource unavailability
ICU admission	6	The clinical condition has deteriorated	NGT/urinary catheter inserted
IVIg Initiation	9	Progressive bulbar weakness (dysphagia/drooling), urinary retention	IVIg 2 g/kg started (over 5 days); gabapentin 300 mg TDS
Symptom peak	8–14	Maximal paralysis	MRC scale = 10 Single breath count was not done due to dysarthria
Clinical improvement begins	15	Audible sounds; reduced drooling; sips of water; minimal limb strength improvement	Transferred to general medical ward; gabapentin and labetalol continued with lower doses
Clinical improvement continued	26	Stands with support; independent voiding; improved speech; oral intake resumed	Discharged
Follow‐up (full recovery of power in all extremities)	44	Normal strength/reflexes/sensation; resumed farming activities	Confirmed return to pre‐event functional status
Follow‐up (complete recovery)	54	He had difficulty articulating the “P” sound, which normalized on the 54th day; he articulated the “P” sound as an “f” sound, likely due to a disturbance in the innervation of the orbicularis oris muscle	

## 5 Discussion

Guillain–Barré syndrome is a rare yet potentially life‐threatening immune‐mediated disorder affecting peripheral nerves and nerve roots, typically triggered by infections [2]. While the exact immunopathological mechanism remains unclear, molecular mimicry—wherein pathogen‐derived antigens elicit cross‐reactive antibodies targeting gangliosides—is implicated in its pathogenesis [3].

GBS is predominantly post‐infectious, with two‐thirds of patients reporting antecedent gastrointestinal or respiratory symptoms [4]. The most frequently identified pathogens include *C. jejuni* (32%), cytomegalovirus (13%), Epstein–Barr virus (10%), and *Mycoplasma pneumoniae* (5%) [5]. In this case, the patient developed GBS 5 days after *P. vivax* malaria, a rare association documented only sporadically in the literature [5, 6]. GBS can also follow non‐infectious insults; for example, Yilmaz et al. [7] and his colleagues described the first reported case of GBS occurring after a craniocerebral gunshot injury. Such reports illustrate that diverse triggers—including trauma or surgery—can, in rare instances, initiate the immune cascade leading to GBS.

To our knowledge, Guillain–Barré syndrome (GBS) following malaria is a rare occurrence, with only 53 documented cases in the English literature, including the present case (Table 2). The average patient age was 32.8 years (range: 8–63), with a male predominance (30 patients, 56.6%) compared to females (23 patients, 43.4%). Among these, *Plasmodium falciparum* accounted for 31 cases (58.5%), *P. vivax* for 21 (39.6%), and mixed infection for one (1.9%). The mean interval between fever onset and paralysis was 6.8 days (range: 3–42 days). Of the 50 patients with documented respiratory status, 12 (24%) developed respiratory paralysis. Among those with respiratory paralysis, 9 patients were infected with *P. falciparum*, of whom 8 succumbed, while 1 survived after receiving intravenous immunoglobulin (IVIG). Additionally, 3 patients with respiratory paralysis were infected with *P. vivax*, with 2 fatalities and 1 survivor who was treated with IVIG [1, 8, 9, 10, 11, 12, 13, 14, 15, 16, 17, 18, 19, 20, 21, 22, 23, 24, 25].

**TABLE 2:** Reports of GBS following malaria in the English literature.

S. no.	Age	Sex	Country/year	Type of plasmodium species	Latency between fever and paralysis	AMT	Duration of paralytic illness	Peak muscle paralysis	NCS	Respiratory failure	Immuno‐modulatory therapy	Outcome (weakness)	References
1	15	Male	India/1980	PV	14	Chloroquine	25	NR	DM	No	Steroid	R	[8]
2	28	Male	India/1980	PV	11	Chloroquine	32	NR	DM	No	None	R	[8]
3	38	Female	India/1985	PF	42	Chloroquine	NR	NR	DM	Yes	Steroid	D	[9]
4	48	Male	India/1985	PF	13	None	NR	NR	DM	Yes	Steroid	D	[9]
5	63	Male	Brazil/1985	PF	21	Pyra/sulfa	NR	NR	DM	Yes	NR	D	[10]
6	32	Male	India/1986	PF	5	None	42	NR	DM	No	None	R	[11]
7	45	Male	Sri Lanka/1992	PF	5	Chloroquine	42	NR	DM	No	None	R	[12]
8	35	Female	India/1996	PF	8	Chloroquine	NR	NR	DM	No	None	R	[13]
9	30	Female	India/1997	PF	16	Quinine	26	NR	DM	No	None	R	[14]
10	47	Male	India/1999	PF	15	None	28	NR	DM	Yes	IVIG	R	[15]
11	24	Male	India/1999	PV	12	Chloroquine	42	NR	DM	No	None	R	[16]
12	28	Female	Thailand/2001	PF	10	Quinine	NR	NR	Mixed	No	Plasma	R	[17]
13	16	Female	Sudan/2002	PF	5	Chloroquine	NR	10	DM	Yes	NR	D	[18]
14	30	Female	Sudan/2002	PF	30	Chloroquine	NR	2	DM	No	NR	R	[18]
15	10	Male	Sudan/2002	PF	7	Chloroquine	NR	3	DM	Yes	NR	D	[18]
16	38	Female	Sudan/2002	PF	15	Chloroquine	NR	15	DM	No	NR	R	[18]
17	35	Male	Sudan/2002	PF	13	Chloroquine	NR	6	DM	Yes	NR	D	[18]
18	23	Male	Sudan/2002	PF	3	Chloroquine	NR	10	DM	No	NR	R	[18]
19	13	Female	Sudan/2002	PF	10	Chloroquine	NR	12	DM	No	NR	R	[18]
20	34	Female	Sudan/2002	PF	10	Chloroquine	NR	11	DM	No	NR	R	[18]
21	54	Male	Sudan/2002	PF	12	Chloroquine	NR	6	DM	Yes	NR	D	[18]
22	48	Male	Sudan/2002	PF	11	Chloroquine	NR	12	DM	No	NR	R	[18]
23	60	Male	India/2003	PF	7	Quinine	2	2	None	NR	None	R	[19]
24	39	Male	India/2004	PV	11	Chloroquine	30	3	Mixed	Yes	IVIG	R	[20]
25	25	Male	India/2009	PF	4	NR	30	NR	Mixed	NR	NR	R	[7]
26	8	Female	India/2011	PF	30	Artesunate	NR	NR	Mixed	No	IVIG	R	[21]
27	60	Female	India/2011	PV	7	Chloroquine	NR	NR	Mixed	NR	Plasma	R	[22]
28	36	Male	India/2012	PF	5–6	Quinine	10	NR	DM	No	None	R	[23]
29	26	Female	Malawi/2014	PF	15	Coartum	21	5	DM	No	None	R	[23]
30	30	NR	India/2015	PF&PV	3	Artesunate	13	NR	Axonal	No	None	R	[24]
31	51	Male	Togo/2022	PF	9	Artesunate	NR	NR	Axonal	No	IVIG	R	[25]
32	22	Female	Pakistan/2024	PV	5	Yes	NR	10	Axonal	Yes	Plasma	D	[1]
33	42	Male	Pakistan/2024	PV	6	Yes	NR	10	Axonal	No	None	R	[1]
34	42	Female	Pakistan/2024	PV	8	Yes	NR	14	Axonal	No	None	R	[1]
35	41	Male	Pakistan/2024	PV	5	Yes	NR	12	Axonal	No	None	R	[1]
36	21	Female	Pakistan/2024	PF	6	Yes	NR	14	DM	Yes	Plasma	D	[1]
37	20	Female	Pakistan/2024	PF	4	Yes	NR	10	Axonal	No	None	R	[1]
38	38	Female	Pakistan/2024	PV	5	Yes	NR	11	Axonal	No	None	R	[1]
39	20	Female	Pakistan/2024	PV	9	Yes	NR	21	Axonal	No	Plasma	R	[1]
40	12	Male	Pakistan/2024	PF	5	Yes	NR	10	Axonal	No	None	R	[1]
41	28	Male	Pakistan/2024	PF	8	Yes	NR	14	Axonal	No	None	R	[1]
42	45	Female	Pakistan/2024	PV	10	Yes	NR	20	Axonal	No	Plasma	R	[1]
43	30	Female	Pakistan/2024	PV	8	Yes	NR	18	DM	No	None	R	[1]
44	38	Male	Pakistan/2024	PV	8	Yes	NR	16	Axonal	Yes	Plasma	D	[1]
45	25	Male	Pakistan/2024	PF	6	Yes	NR	14	Axonal	No	None	R	[1]
46	25	Male	Pakistan/2024	PV	5	Yes	NR	10	Axonal	No	None	R	[1]
47	35	Female	Pakistan/2024	PV	7	Yes	NR	14	Axonal	No	None	R	[1]
48	40	Male	Pakistan/2024	PV	8	Yes	NR	20	Axonal	No	None	R	[1]
49	35	Male	Pakistan/2024	PV	5	Yes	NR	19	Axonal	No	None	R	[1]
50	51	Male	Pakistan/2024	PV	8	Yes	NR	21	Axonal	No	Plasma	R	[1]
51	23	Female	Pakistan/2024	PV	5	Yes	NR	18	Axonal	No	None	R	[1]
52	16	Female	Ethiopia/2025	PF	6	Artesunate	30	3	None	No	None	R	[8]
53	21	Male	Ethiopia/2025	PV	5	Coartum	54	9	None	No	IVIG	R	Our

Abbreviations: Coartum, artemether + lumefantrine; D, death; DM, demyelinating; NR, not reported; PF, *Plasmodium falciparum*; *PV*, *Plasmodium vivax*; Pyra/sulfa, pyramethamine/sulfadoxine; R, recovered.

Although *P. vivax* is less likely than *P. falciparum* to cause cerebral malaria, it can still trigger GBS via immune mechanisms. The parasite's asexual blood stage releases cytokines and other mediators that may induce an autoimmune attack on peripheral nerves. Molecular mimicry and cross‐reactive antibodies (as in classic post‐infectious GBS) have been proposed [26].

Clinically, GBS presents as acute, progressive flaccid paralysis with hyporeflexia or areflexia, often accompanied by sensory disturbances. Cerebrospinal fluid (CSF) analysis typically reveals albuminocytologic dissociation (elevated protein with normal cell count), a hallmark of GBS (2,4). Electrodiagnostic studies, such as nerve conduction studies (NCS), are valuable for confirming atypical cases of Guillain–Barré Syndrome (GBS) and classifying it into axonal, demyelinating, or mixed variants [2]. In this case, NCS was not performed. However, GBS remains primarily a clinical diagnosis, reliably established through a detailed history and physical examination [27]. The diagnosis in our patient was supported by the classic presentation of ascending flaccid paralysis with areflexia, corroborated by cerebrospinal fluid (CSF) analysis showing albuminocytologic dissociation. In our review, among the 48 patients who underwent nerve conduction studies, 25 (51%) demonstrated demyelinating motor neuropathy, 20 (41.7%) had axonal neuropathy, and 3 (6%) exhibited a mixed pattern.

The management of Guillain–Barré Syndrome (GBS) focuses on immunomodulatory therapy, such as intravenous immunoglobulin (IVIg) or plasma exchange, initiated within 2–4 weeks of symptom onset, alongside supportive care [2]. Early immunotherapy is indicated to hasten recovery and minimize complications in moderate to severe GBS cases [26]. Conversely, very mild GBS cases may recover without specific therapy [27]. Supportive care includes gabapentin to alleviate neuropathic pain, a common and often severe symptom in GBS [8], and labetalol to manage autonomic dysfunction, specifically labile blood pressure [8]. These interventions are standard supportive measures to support neurological recovery.

## 6 Conclusion

This case illustrates a rare association between *P. vivax* malaria and GBS. The temporal relationship between malaria resolution and GBS onset suggests a post‐infectious process. Despite the lack of nerve conduction studies, the clinical and CSF findings were diagnostic. Although malaria is a common infection in endemic regions, its association with GBS is scarcely reported in the English literature.

The patient's rapid response to intravenous immunoglobulin (IVIg) and supportive care underscores the importance of early diagnosis and treatment in improving outcomes. This case adds to the limited literature on malaria‐associated GBS and highlights the need for increased awareness of this complication in malaria‐endemic areas. Further studies are needed to explore the mechanisms underlying this association and optimize management strategies.

## 7 Limitations

- *Nerve conduction studies*: Not performed due to resource constraints, which could have provided additional diagnostic confirmation and subclassifying GBS.
- *Serological testing*: Limited testing for other potential triggers of GBS (e.g., *C. jejuni* , viral infections).

## Author Contributions

**Hayatu Awel Abdela:** conceptualization, data curation, formal analysis, investigation, writing – original draft, writing – review and editing. **Numeri Husein Kimo:** data curation, software, writing – original draft, writing – review and editing. **Tamirat Godebo Woyimo:** conceptualization, data curation, formal analysis, funding acquisition, investigation, methodology, software, validation, writing – original draft, writing – review and editing.

## Ethics Statement

Ethical clearance was obtained from the Institutional Review Board (IRB) of College of Health Science and Medicine, Wolkite University.

## Consent

Written informed consent was obtained from the patient before starting the data collection process. The confidentiality and privacy of the patient were assured. Neither the data records nor the extracted data were used for any other purpose.

## Conflicts of Interest

The authors declare no conflicts of interest.

## Data Availability

The data that support the findings of this study are available from the corresponding author upon reasonable request.
